# Parental Misidentification and Potential Mismanagement of Dermatophytosis: Insights From a Nationwide Survey of Mothers, United States, 2025

**DOI:** 10.1111/pde.70128

**Published:** 2026-01-23

**Authors:** Jeremy A. W. Gold, Caitlyn Lutfy, Kaitlyn Werner, Shari R. Lipner, Kaitlin Benedict

**Affiliations:** 1 Mycotic Diseases Branch, Division of Foodborne, Waterborne, and Environmental Diseases, Centers for Disease Control and Prevention, Atlanta, Georgia, USA; 2 Outbreak Response and Prevention Branch, Division of Foodborne, Waterborne, and Environmental Diseases, Centers for Disease Control and Prevention, Atlanta, Georgia, USA; 3 Department of Dermatology, Weill Cornell Medicine, New York, New York, USA

**Keywords:** child health, corticosteroids, dermatophytosis, fungal drug resistance, surveys and questionnaires

## Abstract

Information is lacking on parental recognition and management practices for dermatophytosis, a common childhood infection associated with increasing antifungal resistance. We analyzed data from a nationwide survey of US mothers with children < 18 years living at home, using chi-squared tests to compare respondents who indicated they would treat dermatophytosis with over-the-counter corticosteroids versus those who would not. Among the 306 respondents, 47% correctly identified ringworm and 63% misattributed erythema migrans as ringworm; 17% said they would try an over-the-counter corticosteroid cream for suspected dermatophytosis, a practice more frequently reported among respondents who were non-Hispanic Black (29% vs. 11%), Hispanic/Latino (10% vs. 8%), and non-Hispanic multiracial (16% vs. 11%) (*p* = 0.009) and who had a high school education or less (45% vs. 27%, *p* = 0.032). Our study emphasizes the challenges of visually diagnosing dermatophytosis and the importance of early healthcare evaluation to ensure accurate diagnosis and proper treatment.

## 1 | Introduction

Dermatophytosis (ringworm) is a common childhood infection receiving renewed attention because of emerging antifungal-resistant infections [1–3]. Parents are often the first to respond to childhood rashes, but information is lacking on US parental recognition and management practices. We analyzed data from a nationwide survey of US mothers with children < 18 years living at home.

## 2 | Methods

CDC contracted Porter Novelli (PN), a communications firm specializing in social marketing and public health research, to assist with survey development, recruitment strategy, and fielding. We used PN View Moms survey data (https://styles.porternovelli.com/pn-view-panels/). The survey was conducted in English during July 22–25, 2025, using quota sampling based on region, income, and race/ethnicity to achieve a nationwide sample.

Participants reported demographic information, answered questions on treatment and prevention, and attempted to identify images of ringworm rashes among a series of photos of various types of rashes (Table 1, Figure S1). They also indicated actions they would take if they suspected their child had ringworm, factors prompting care seeking, and ringworm prevention measures.

Traditional survey response rates are inapplicable, as response links remain accessible to everyone in the study sample pool. To facilitate rapid fielding, no restrictions on the number of clicks were implemented, so quotas often fill before all respondents could participate. In total, the survey recorded 880 clicks, with 306 completed surveys, 126 incomplete responses, and 448 terminations or no responses. We analyzed the number and frequency of responses for completed surveys. Chi-squared tests compared respondents who indicated they would (vs. would not) treat dermatophytosis with over-the-counter corticosteroid creams.

## 3 | Results

Among the 306 respondents, the median age was 38.5 years (interquartile range: 31.0–45.0). The largest percentage were from the South (37%), followed by the West (26%) (Table 1). Most were non-Hispanic White (61%), with 14% non-Hispanic Black, 11% non-Hispanic Multiracial, and 8% Hispanic/Latino. The highest educational level was a bachelor’s degree or higher for 35%, 34% with some college, and 30% with a high school education or less.

When shown images of common childhood rashes, 47% correctly identified ringworm and 63% misattributed erythema migrans as ringworm; fewer misattributed eczema/psoriasis (25%), hives (3%), or chickenpox (2%) as ringworm (Table 2). When asked what they would do if they believed that their child had ringworm, 82% indicated they would contact their healthcare provider or pediatrician; 31% would try an over-the-counter antifungal cream, 17% would try an over-the-counter corticosteroid cream, and 13% would try natural remedies. Potential over-the-counter corticosteroid cream use (vs. non-use) was more frequently reported among respondents who were Black (29% vs. 11%), Hispanic/Latino (10% vs. 8%), and multiracial (16% vs. 11%) (*p* = 0.009) and who had a high school education or less (45% vs. 27%, *p* = 0.032); other comparisons were non-significant (Table S1).

Respondents indicated they would be most likely to seek medical care for their child’s ringworm if the rash was large or spreading (78%) or appeared infected with redness or swelling (74%). Other triggers included lack of improvement with home treatment (64%), substantial itchiness (54%), and rash location on the scalp or face (52%). For prevention, the most frequently reported strategies were keeping children’s clothing clean (62%), showering after sports (62%), and keeping skin clean and dry (61%).

## 4 | Discussion

In this nationwide survey of US mothers, under half of respondents (47%) correctly identified dermatophytosis from an image, highlighting the difficulty of visually diagnosing rashes and the importance of seeking early healthcare for accurate diagnosis and treatment. Nearly one-fifth (17%) reported they would use an over-the-counter corticosteroid for ringworm, which can worsen fungal infections [4]. This response was more frequent among Black respondents and those with lower educational attainment, groups that might be disproportionately affected by dermatophytosis [5–7].

Reassuringly, most respondents (78%) said they would consult a healthcare provider or pediatrician if a rash was spreading or appeared infected; however, only about half would seek care if it was on the face or scalp. Localized, non-severe tinea faciei can often be treated with topical antifungals, but tinea capitis requires evaluation by a healthcare provider and treatment with systemic antifungals to ensure hair shaft penetration [1, 4]. Delayed care seeking could potentially contribute to prolonged illness, complications, and preventable transmission.

## 5 | Limitations

Important limitations include the lack of diversity in survey images, lack of assessment of participants’ understanding that “ringworm” is fungal, the opt-in and English-language–only design limiting generalizability, and the small sample size precluding multivariable analyses.

## 6 | Conclusion

Our findings suggest that focused education might help parents determine when it is necessary to seek care for suspected dermatophytosis and could emphasize the importance of avoiding over-the-counter topical corticosteroids for such conditions.

## Supplementary Material

Supplementary material

Additional supporting information can be found online in the Supporting Information section. Figure S1: Images used for survey about ringworm in children. Table S1: Characteristics of participants reporting they would vs. would not treat ringworm with an over-the-counter (OTC) corticosteroid cream.

## Figures and Tables

**TABLE 1 |:** Survey respondents’ demographic characteristics (*N* = 306).

Response	No. (%)
Age group, years	
18–29	62 (20)
30–39	97 (32)
40–49	105 (34)
50+	42 (14)
Region	
Northeast	49 (16)
Midwest	64 (21)
South	113 (37)
West	80 (26)
Marital status	
Married or living with partner	208 (68)
Single, separated, divorced, widowed	98 (32)
Own or rent dwelling	
Own	167 (55)
Rent	127 (42)
Live with others at no cost	12 (4)
Highest level of formal education completed	
High school or less	93 (30)
Some college	105 (34)
Bachelor’s degree or higher	108 (35)
Employment status	
Working full or part time or student	215 (70)
Not working	91 (30)
Total household income before taxes	
Less than $75,000	165 (54)
$75,000 or more	141 (46)
Race/ethnicity	
African/African American/Black, non-Hispanic	44 (14)
Asian, non-Hispanic	7 (2)
Hispanic/Latinx	26 (8)
Multiple race, non-Hispanic	35 (11)
Other race/ethnicity, non-Hispanic	7 (2)
White, non-Hispanic	187 (61)
Community type	
Urban	76 (25)
Suburban	160 (52)
Rural	70 (23)
Political affiliation	
Republican	82 (27)
Democrat	88 (29)
Independent	87 (28)
Something else	9 (3)
Not sure	29 (9)
Decline to answer	11 (4)
Childrens’ ages	
Infant 1–3 weeks	4 (1)
Infant 4–7 weeks	7 (2)
Infant 2 months to toddler (2 months to 2 years)	74 (24)
Preschool-aged (3–5 years)	92 (30)
Elementary school-aged (6–10 years)	136 (44)
Middle school-aged (11–13 years)	101 (33)
High school-aged (14–17 years)	114 (37)
Total children in household	
1	114 (37)
2	102 (33)
3	48 (16)
4 or more	42 (14)

**TABLE 2 |:** Survey respondents’ answers to questions about ringworm (*N* = 306).

	No. (%)
Of the rashes pictured, which are images of ringworm?	
A (hives)	10 (3)
B (ringworm)	145 (47)
C (eczema or psoriasis)	75 (25)
D (erythema migrans)	193 (63)
E (chicken pox)	5 (2)
None of these [EXCLUSIVE]	1 (0)
I do not know [EXCLUSIVE]	20 (7)
If you thought your child had ringworm, what would you do?	
Treat it with over-the-counter antifungal cream	96 (31)
Treat it with over-the-counter steroid cream (e.g., cortisone)	51 (17)
Treat it with a natural remedy (e.g., tea tree oil, apple cider vinegar)	39 (13)
Contact your healthcare provider/pediatrician	250 (82)
Search online for advice	74 (24)
Search using ChatGPT or another AI service for advice	26 (8)
Ask a friend or a family member for advice	55 (18)
Wait and see if it gets worse before acting	10 (3)
I do not know what ringworm is [EXCLUSIVE]	3 (1)
None of these [EXCLUSIVE]	3 (1)
Which of the following would make you more likely to seek medical care for your child’s ringworm?	
The ringworm rash is large or spreading	240 (78)
The ringworm rash is very itchy	164 (54)
The ringworm rash is on their scalp or face	158 (52)
The ringworm rash does not improve with home treatment	196 (64)
The ringworm rash looks infected (redness or swelling)	225 (74)
None of these [EXCLUSIVE]	12 (4)
What do you do to prevent ringworm in your child(ren)? (*n* = 303)	
Educate my child about avoiding contact with infected people or animals	157 (52)
Keep my child’s clothing and other personal items clean and dry (shoes, gym bags)	189 (62)
Discourage walking barefoot in public places (pools, locker rooms)	169 (56)
Have my child take a shower after sports practices, games, or matches	187 (62)
Discourage sharing clothing, towels, sheets, or other personal items	167 (55)
Make sure my child’s skin is kept clean and dry	184 (61)
Make sure my child’s fingernails and toenails are kept short and clean	158 (52)
I am not concerned about ringworm [EXCLUSIVE]	25 (8)
I do not try to prevent ringworm in my children [EXCLUSIVE]	7 (2)

## Data Availability

The data used in this analysis cannot be shared publicly because the CDC licensed the data from Porter Novelli. The data are owned by Porter Novelli, a third party, and the authors do not have permission to share the data. The data underlying the results presented in the study are available from Porter Novelli (https://www.porternovelli.com/).
