# Supplementary material for: Parental Misidentification and Potential Mismanagement of Dermatophytosis: Insights From a Nationwide Survey of Mothers, United States, 2025
Source: Pediatr Dermatol. Author manuscript; Available in PMC 2026 Mar 5. (PMC12958439; doi:10.1111/pde.70128)
Supplement: Supplementary material [file NIHMS2145077-supplement-Supplementary_material.docx]

**Supplementary Figure.** Images used for survey about ringworm in children

1. Hives

**
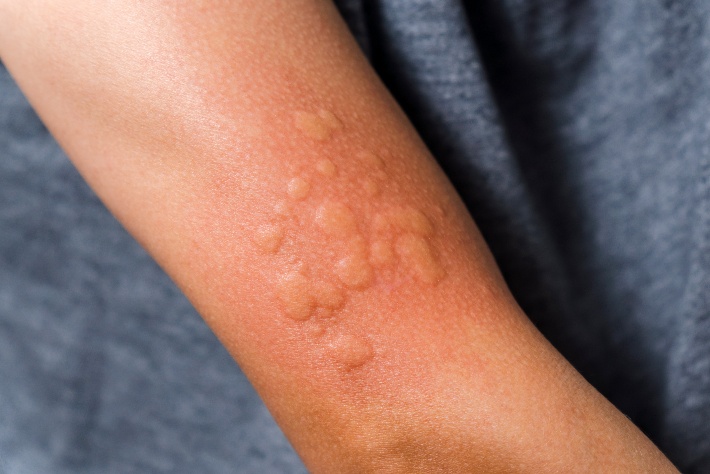
**

1. Ringworm

**
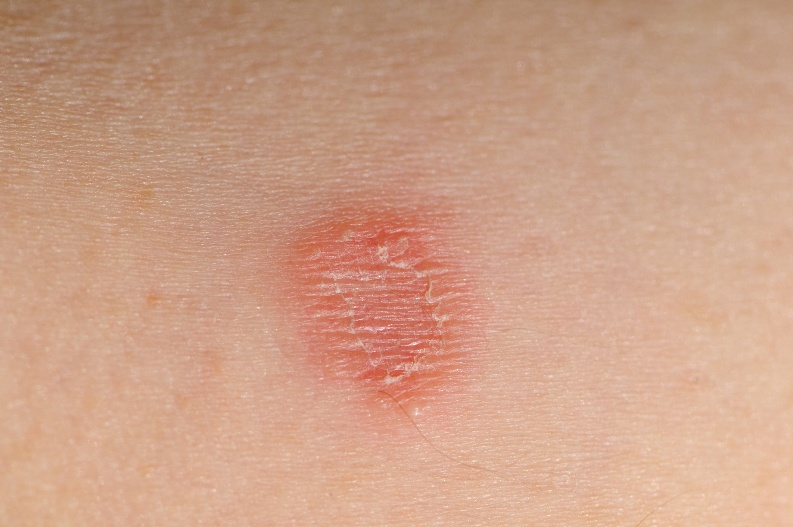
**

1. Eczema or psoriasis


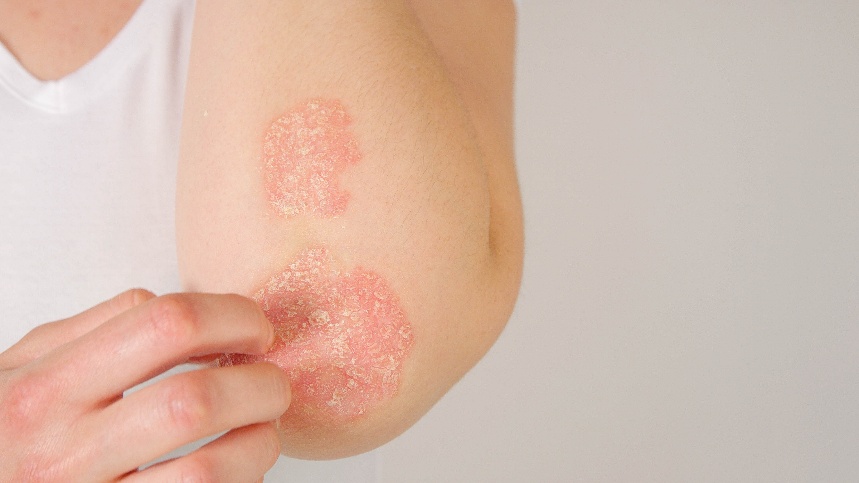


1. Erythema migrans

**
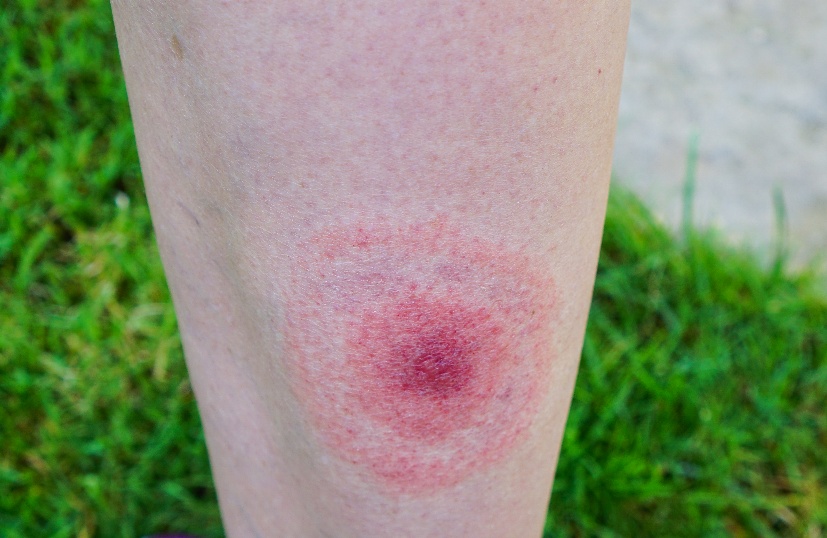
**

1. **
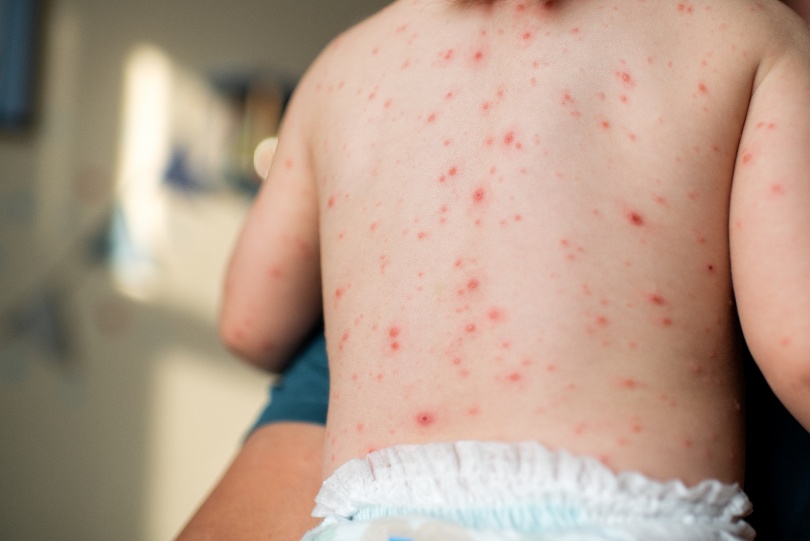
**Chicken pox

*Credit: Getty Images*

| **Supplemental Table. Characteristics of participants reporting they would vs. would not treat ringworm with an over-the-counter (OTC) corticosteroid cream** | | | | | |
| --- | --- | --- | --- | --- | --- |
| **Characteristic** | **Treat with OTC steroid cream** | | **Not treat with OTC steroid cream** | |  |
|  | **n=51** | **col %** | **n=255** | **col %** | **p-value** |
| **Age group, years** | |  |  |  | 0.104 |
| 18 to 29 | 16 | 31% | 46 | 18% |  |
| 30 to 39 | 17 | 33% | 80 | 31% |  |
| 30 to 49 | 14 | 27% | 91 | 36% |  |
| 50+ | 4 | 8% | 38 | 15% |  |
| **Region** |  |  |  |  | 0.605 |
| Northeast | 7 | 14% | 42 | 16% |  |
| Midwest | 10 | 20% | 54 | 21% |  |
| South | 23 | 45% | 90 | 35% |  |
| West | 11 | 22% | 69 | 27% |  |
| **Marital status** | |  |  |  | 0.381 |
| Married or living with partner | 32 | 63% | 176 | 69% |  |
| Single, separated, divorced, widowed | 19 | 37% | 79 | 31% |  |
| **Own or rent dwelling** | | |  |  | 0.278 |
| Own | 24 | 47% | 143 | 56% |  |
| Rent | 26 | 51% | 101 | 40% |  |
| Live with others at no cost | 1 | 2% | 11 | 4% |  |
| **Highest level of formal education completed** | | | | | 0.032 |
| High school or less | 23 | 45% | 70 | 27% |  |
| Some college | 16 | 31% | 89 | 35% |  |
| Bachelor's degree or higher | 12 | 24% | 96 | 38% |  |
| **Employment status** | |  |  |  | 0.288 |
| Working full or part time or student | 39 | 76% | 176 | 69% |  |
| Not working | 12 | 24% | 79 | 31% |  |
| **Total household income before taxes** | | | |  | 0.166 |
| Less than $75,000 | 32 | 63% | 133 | 52% |  |
| $75,000 or more | 19 | 37% | 122 | 48% |  |
| **Race/ethnicity** | |  |  |  | 0.009 |
| African/African American/Black, non-Hispanic | 15 | 29% | 29 | 11% |  |
| Asian, non-Hispanic | 0 | 0% | 7 | 3% |  |
| Hispanic/Latinx | 5 | 10% | 21 | 8% |  |
| Multiple race, non-Hispanic | 8 | 16% | 27 | 11% |  |
| Other race/ethnicity, non-Hispanic | 1 | 2% | 6 | 2% |  |
| White, non-Hispanic | 22 | 43% | 165 | 65% |  |
| **Community type** | |  |  |  | 0.084 |
| Urban | 18 | 35% | 58 | 23% |  |
| Suburban | 26 | 51% | 134 | 53% |  |
| Rural | 7 | 14% | 63 | 25% |  |
| **Political affiliation** | |  |  |  | 0.666 |
| Republican | 14 | 27% | 68 | 27% |  |
| Democrat | 19 | 37% | 69 | 27% |  |
| Independent | 13 | 25% | 74 | 29% |  |
| Something else | 1 | 2% | 8 | 3% |  |
| Not sure | 3 | 6% | 26 | 10% |  |
| Decline to answer | 1 | 2% | 10 | 4% |  |
| **Childrens' ages** | |  |  |  |  |
| Infant 1-3 weeks | 1 | 2% | 3 | 1% | 0.520 |
| Infant 4-7 weeks | 1 | 2% | 6 | 2% | 0.864 |
| Infant 2 months to toddler (2 months-2 years) | 14 | 27% | 60 | 24% | 0.551 |
| Preschool-aged (3-5 years) | 17 | 33% | 75 | 29% | 0.577 |
| Elementary school-aged (6-10 years) | 27 | 53% | 109 | 43% | 0.181 |
| Middle school-aged (11-13 years) | 10 | 20% | 91 | 36% | 0.026 |
| High school-aged (14-17 years) | 20 | 39% | 94 | 37% | 0.751 |
| **Total children in household** | | |  |  | 0.763 |
| 1 | 17 | 33% | 97 | 38% |  |
| 2 | 16 | 31% | 86 | 34% |  |
| 3 | 9 | 18% | 39 | 15% |  |
| 4 or more | 9 | 18% | 33 | 13% |  |
